# Choroid plexus volume as a novel candidate neuroimaging marker of the Alzheimer’s continuum

**DOI:** 10.1186/s13195-024-01520-w

**Published:** 2024-07-03

**Authors:** Jiwei Jiang, Zhizheng Zhuo, Anxin Wang, Wenyi Li, Shirui Jiang, Yunyun Duan, Qiwei Ren, Min Zhao, Linlin Wang, Shiyi Yang, Maher Un Nisa Awan, Yaou Liu, Jun Xu

**Affiliations:** 1 Beijing Tiantan Hospital, Capital Medical University, Beijing, China; 2 National Clinical Research Center for Neurological Diseases, Beijing, China; 3 The Affiliated Hospital of Yunnan University, Kunming, China

**Keywords:** Choroid plexus, Alzheimer’s disease, Mediation analysis, ROC curve, Amyloid-beta, Longitudinal studies

## Abstract

**Background:**

Enlarged choroid plexus (ChP) volume has been reported in patients with Alzheimer’s disease (AD) and inversely correlated with cognitive performance. However, its clinical diagnostic and predictive value, and mechanisms by which ChP impacts the AD continuum remain unclear.

**Methods:**

This prospective cohort study enrolled 607 participants [healthy control (HC): 110, mild cognitive impairment (MCI): 269, AD dementia: 228] from the Chinese Imaging, Biomarkers, and Lifestyle study between January 1, 2021, and December 31, 2022. Of the 497 patients on the AD continuum, 138 underwent lumbar puncture for cerebrospinal fluid (CSF) hallmark testing. The relationships between ChP volume and CSF pathological hallmarks (Aβ_42_, Aβ_40_, Aβ_42/40_, tTau, and pTau_181_), neuropsychological tests [Mini-Mental State Examination (MMSE), Montreal Cognitive Assessment (MoCA), Neuropsychiatric Inventory (NPI), and Activities of Daily Living (ADL) scores], and multimodal neuroimaging measures [gray matter volume, cortical thickness, and corrected cerebral blood flow (cCBF)] were analyzed using partial Spearman’s correlation. The mediating effects of four neuroimaging measures [ChP volume, hippocampal volume, lateral ventricular volume (LVV), and entorhinal cortical thickness (ECT)] on the relationship between CSF hallmarks and neuropsychological tests were examined. The ability of the four neuroimaging measures to identify cerebral Aβ_42_ changes or differentiate among patients with AD dementia, MCI and HCs was determined using receiver operating characteristic analysis, and their associations with neuropsychological test scores at baseline were evaluated by linear regression. Longitudinal associations between the rate of change in the four neuroimaging measures and neuropsychological tests scores were evaluated on the AD continuum using generalized linear mixed-effects models.

**Results:**

The participants’ mean age was 65.99 ± 8.79 years. Patients with AD dementia exhibited the largest baseline ChP volume than the other groups (*P* < 0.05). ChP volume enlargement correlated with decreased Aβ_42_ and Aβ_40_ levels; lower MMSE and MoCA and higher NPI and ADL scores; and lower volume, cortical thickness, and cCBF in other cognition-related regions (all *P* < 0.05). ChP volume mediated the association of Aβ_42_ and Aβ_40_ levels with MMSE scores (19.08% and 36.57%), and Aβ_42_ levels mediated the association of ChP volume and MMSE or MoCA scores (39.49% and 34.36%). ChP volume alone better identified cerebral Aβ_42_ changes than LVV alone (AUC = 0.81 vs. 0.67, *P* = 0.04) and EC thickness alone (AUC = 0.81 vs.0.63, *P* = 0.01) and better differentiated patients with MCI from HCs than hippocampal volume alone (AUC = 0.85 vs. 0.81, *P* = 0.01), and LVV alone (AUC = 0.85 vs.0.82, *P* = 0.03). Combined ChP and hippocampal volumes significantly increased the ability to differentiate cerebral Aβ_42_ changes and patients among AD dementia, MCI, and HCs groups compared with hippocampal volume alone (all *P* < 0.05). After correcting for age, sex, years of education, *APOE* ε4 status, eTIV, and hippocampal volume, ChP volume was associated with MMSE, MoCA, NPI, and ADL score at baseline, and rapid ChP volume enlargement was associated with faster deterioration in NPI scores with an average follow-up of 10.03 ± 4.45 months (all *P* < 0.05).

**Conclusions:**

ChP volume may be a novel neuroimaging marker associated with neurodegenerative changes and clinical AD manifestations. It could better detect the early stages of the AD and predict prognosis, and significantly enhance the differential diagnostic ability of hippocampus on the AD continuum.

**Supplementary Information:**

The online version contains supplementary material available at 10.1186/s13195-024-01520-w.

## Background

Alzheimer’s disease (AD) is a complex, heterogeneous disorder with multifaceted neuropathological characteristics, including the widespread accumulation of beta-amyloid (Aβ) plaques, neurofibrillary tangles, and blood–brain barrier (BBB) dysfunction, which significantly complicate the diagnosis and treatment of AD in clinical practice [1, 2]. The proposed updated amyloid, tau, neurodegeneration [AT(N)] research framework accelerates the diagnosis of the AD continuum by 6–18 years compared with previous recommendations [3, 4]. However, the high costs and invasiveness of cerebrospinal fluid (CSF) testing and positron emission tomography limit their usefulness for detecting pathological hallmarks in clinical practice [5, 6]. Traditional structural imaging is convenient and inexpensive, but lacks the molecular specificity to directly determine the neural source of volume or thickness loss [7, 8]. Historically, the structural indicators of AD neuropathology that are considered optimal, including cortical and hippocampal atrophy and ventricular dilation, often occur in normal aging processes and other neurodegenerative diseases [9]. Therefore, they cannot serve as stand-alone indicators for early diagnosis in the AD continuum [10, 11]. There is an urgent need to identify a non-invasive, reliable neuroimaging marker associated with the multiomic features of the AD continuum to further elucidate the complexity and heterogeneity of AD pathogenesis. Furthermore, we need to examine the value of combining multiple structural neuroimaging measures in enabling early detection and development of treatment strategies for patients across the AD continuum.

The choroid plexus (ChP) is an important multifunctional structure involved in CSF secretion, immune surveillance, and BBB preservation, all of which are closely linked to multiple pathological pathways in AD [12, 13]. Impaired ChP function has been recently discovered in patients in the AD continuum, including reduced clearance of CSF Aβ and Tau [14, 15]. Preliminary studies revealed that aging-related CSF changes were associated with significant ChP remodeling, which was correlated with cognitive performance in patients with AD [16, 17]. Therefore, ChP may represent a hitherto overlooked but important candidate neuroimaging marker to track the onset and development of AD. The existing evidence only presents preliminary differences in ChP volume during different stages of the AD continuum at baseline [18, 19]. The association between ChP volume and clinical manifestations, multifunctional brain structures, and other multiomic neurodegenerative characteristics of the AD continuum remains unclear: the exact role and mechanism by which the ChP is involved in the onset and development of AD are unknown.

In this study, we investigated the associations of ChP volume with pathological CSF hallmarks, neuropsychological tests, and multimodal neuroimaging measures; and analyzed the diagnostic accuracy of ChP volume to identify cerebral pathological deposition, and differentiate among patients with AD dementia, mild cognitive impairment (MCI), and healthy controls (HCs). Importantly, we examined the longitudinal association between baseline ChP volume and subsequent clinical progression of the AD continuum. Our principal hypotheses were that (1) enlarged ChP volume was highly associated with the severity of pathological CSF hallmarks, neuropsychological tests, and the structure and perfusion of other cognition-related brain regions; (2) ChP volume could mediate the association between CSF hallmarks and neuropsychological tests on the AD continuum; (3) ChP volume could identify the absence/presence of cerebral Aβ_42_ deposition, and differentiate among stages along the Alzheimer’s continuum; and (4) increased ChP volume would accelerate the deterioration in the clinical presentations.

## Methods

### Participants

This study used data prospectively collected from the Chinese Imaging, Biomarkers, and Lifestyle (CIBL) study between January 1, 2021, and December 31, 2022. The CIBL study [20, 21] was approved by the Institutional Review Board of Capital Medical University, Beijing Tiantan Hospital (Beijing, China; KY-2021-028-01) and registered at Chictr.org.cn (ChiCTR2100049131). All participants or their caregivers provided written informed consent. The AD continuum included patients with MCI and AD dementia who met the 2011 or 2018 National Institute on Aging Alzheimer’s Association workgroup diagnostic criteria [3, 22]. We also enrolled HCs aged ≥ 50 years without subjective or objective memory impairment or history of neurological diseases. Trained research coordinators interviewed patients with MCI/AD personally at approximately 6-month intervals until June 30, 2023. A detailed flowchart of the eligibility criteria at baseline and follow-up is presented in Supplementary Fig. 1. This study conformed to the Strengthening the Reporting of Observational Studies in Epidemiology guidelines (Supplementary Material S01).

### Clinical measures and neuropsychological tests

Data on age, sex, years of education, body mass index (BMI), apolipoprotein E allele 4 (*APOE* ε4) status; medical history of hypertension, diabetes mellitus, stroke, coronary heart disease, and hyperlipidemia; and smoking and alcohol consumption habits were collected. Each participant underwent comprehensive neuropsychological battery testing, including the Mini-Mental State Examination (MMSE) [23], Montreal Cognitive Assessment (MoCA) [24], Neuropsychiatric Inventory (NPI) [25], and Activities of Daily Living (ADL) (Supplementary Material S02).

### CSF biomarker collection and measurements

Patients on the AD continuum who agreed to undergo lumbar puncture fasted overnight for a minimum of 6 h. Sterile polypropylene tubes were used to collect CSF and inverted gently to disrupt potential gradient effects, centrifuged at 1500 r/min to separate any cellular debris, and stored at − 80 °C. The CSF levels of Aβ_42_ and Aβ_40_, Aβ_42/__40_ ratio, total Tau protein (tTau), and phosphorylated Tau 181 (pTau_181_) at baseline were measured using the direct enzyme-linked immunosorbent assay (ELISA; Beijing Hightrust Diagnostics, Co, Ltd, China). The resulting optical densities were measured at 450 nm using an RT-6100 Auto Analyzer micro-ELISA plate reader (Rayto Life and Analytical Sciences Co., Ltd. Shenzhen, China). CSF biomarker levels (pg/mL) were calculated using standard curves from the assay kits based on the values obtained from the optical readings. We defined either Aβ_42_ < 650 pg/mL or Aβ_42/40_ ratio ≤ 0.064 as Aβ_42_ positivity.

### Brain multimodal neuroimaging

Structural three-dimensional T1-weighted imaging (3D-T1WI) and 7-delay pseudo-continuous arterial spin labeling (pCASL) were performed using a 3.0-T magnetic resonance scanner (SIGNA Premier; GE Healthcare, Milwaukee, WI, USA) with a 48-channel head coil. Scan parameters of the 3D-T1WI were as follows: repetition time, 7.3 ms; echo time, 3.0 ms; inversion time, 450 ms; flip angle, 12°; field of view, 256 mm*×*256 mm; acquisition matrix, 256* × *256; slice thickness, 1.0 mm; slice number, 176; and scan time, 4 min 56 s. The gray matter (GM) volume and cortical thickness of well-documented cognition-related brain regions were quantified using FreeSurfer (version 6.0, http://surfer.nmr.mgh.harvard.edu/) [26], including the cortical volume, subcortical GM volume, lateral ventricular volume (LVV), hippocampal volume, white matter hypointensity (WMH) volume, and estimated total intracranial volume (eTIV), and the average whole cortical thickness, entorhinal cortical thickness (ECT), middle temporal cortical thickness, and parahippocampal cortical thickness [27–29]. The regional corrected cerebral blood flow (cCBF), including the whole brain, hippocampus, thalamus, middle cingulate cortex, posterior cingulate cortex, middle frontal cortex, superior temporal cortex, precuneus, and angular gyrus [30], was extracted from the preprocessed pCASL image using the corrected arterial transit time values based on the third version of the automated anatomical labelling atlas.

The ChP was segmented based on 3D-T1WI using a deep learning algorithm developed by the authors using 3D nnU-Net as previously described [31]. The ChP of 90 HCs were imaged using 3T scanners [GE Premier, USA (*n* = 30), Philips CX, The Netherlands (*n* = 30), and Siemens Prisma, Germany (*n* = 30)] at our hospital, and manually labelled by a junior neuroradiologist (Zhuo Z., with 5 years of experience in neuroradiology) and a senior neuroradiologist (Duan Y., with 15 years of experience). Subsequently, a senior radiologist with 20 years of experience (Liu Y.) performed a manual visual check and modification (if necessary) for segmentation quality control. A subset of 60 randomly selected disease cases was utilized for training the 3D nnU-Net, and the remaining 30 cases were used for testing the trained model (Dice score = 0.8; *P* < 0.001). Finally, the resulting deep learning model was used to segment all the T1-weighted images used in this study, and the ChP volume was subsequently calculated. Supplementary Material S03 details the multimodal neuroimaging parameters and processing.

All automatic segmentation was performed by a junior neuroradiologist (Zhuo Z.), and the preliminary segmentation results were reviewed and modified for segmentation quality control by a senior neuroradiologist (Duan Y.). All the final automatic segmentation results were assessed and approved by another senior neuroradiologist (Liu Y.) who was blinded to all clinical data. For quality control of ChP segmentation, we focused on the following features by visual inspection: (1) the location of segmentation; (2) the distribution of the ChP; and (3) the morphology of the segmentation. If the ChP segmentation was outside the ventricular areas, the segmentation involved non-ChP tissues, or the 3D rendered images of ChP morphology appeared odd, manual correction was conducted using ITK- SNAP software. For quality control of brain tissue segmentation using FreeSurfer, the initial detection of inaccurate segmentations was based on Euler numbers [32], and subsequent visual checks and modification were performed using FSLeyes according to the standard operating procedure of the FreeSurfer’s quality control guidelines (https://surfer.nmr.mgh.harvard.edu/fswiki). The detailed data exclusion procedure is presented in eFigure 1.

### Statistical analysis

Categorical variables are expressed as raw numbers (percentages) and assessed using the χ^2^ test. Normally distributed continuous variables are presented as the mean [standard deviation (SD)] and evaluated using univariate analysis of variance or independent *t*-tests; skewed data are presented as the median with interquartile range (IQR) and evaluated using the Kruskal–Wallis or Mann–Whitney U test. Post hoc pairwise comparisons were adjusted using Bonferroni correction.

Partial Spearman’s correlation (adjusted for age, sex, years of education, *APOE* ε4 status, and eTIV) was employed to determine the correlation coefficients between both ChP volume and ChP/eTIV, a similar index presented in previous studies [19, 33], and CSF hallmarks (Aβ_42_, Aβ_40_, Aβ_42/40_, tTau, and pTau_181_ levels), neuropsychological tests (MMSE, MoCA, NPI, and ADL scores), and multimodal neuroimaging measures (GM volume, cortical thickness and cCBF of cognition-related brain regions). To estimate the association of ChP/eTIV with these measures, we only adjusted for age, sex, years of education, and *APOE* ε4 status.

Simple mediation models were developed using bootstrapping with 5,000 iterations by establishing three pathways to explain the effects of each neuroimaging measure (ChP volume, hippocampal volume, LVV, or ECT) alone on the association between the CSF hallmarks (Aβ_42_ and Aβ_40_ levels) and neuropsychological tests. Under this framework, the total effect (TE) was divided into natural direct effects (NDEs) and natural indirect effects (NIEs), and the mediation effect was quantified as a percentage (NIE/TE x 100) [34]. The NDEs represent the effect of CSF hallmarks on the neuropsychological tests independent of four neuroimaging measures. The NIEs represent the effect of CSF hallmarks on neuropsychological tests, which could be explained by changes in each neuroimaging measure. In addition, to examine and discuss different potential causal pathways, simple mediation models were also developed by establishing three pathways to explain the effects of CSF hallmarks on the association between ChP volume and the neuropsychological tests. Age, sex, years of education, *APOE* ε4 status, and eTIV at baseline were included as confounders in the mediating analyses based on the results of the above-mentioned univariate analysis.

The area under the receiver operating characteristic (ROC) curve (AUC), sensitivity, and specificity were used to estimate the ability of each neuroimaging measure alone, combined ChP and hippocampal volume, or the combination of the four neuroimaging measures to identify the cerebral Aβ_42_ changes (< 650 pg/mL or not). Furthermore, these factors were also used to differentiate between patients with AD dementia and those with MCI, patients with AD dementia and those with normal cognition, and patients with MCI and those with normal cognition based on binary logistic regression models with adjustment for age, sex, years of education, *APOE* ε4 status, and eTIV. DeLong’s test was performed to statistically compare the AUCs.

Multiple linear regression models were used to determine the relationship of each neuroimaging measure alone and combined ChP and hippocampal volume with the neuropsychological tests in patients across the AD continuum at baseline with adjustment for age, sex, years of education, *APOE* ε4 status, and eTIV. Generalized linear mixed-effects models with a random intercept and slope were utilized to discern the longitudinal association between the rate of change in each neuroimaging measure alone and the rate of change in neuropsychological tests and between the rate of change in the combined ChP and hippocampal volume and the rate of change in neuropsychological tests in patients on the AD continuum during the follow-up period. This model used maximum likelihood estimation, a normal distribution with identity links, and the Kenward–Roger method to approximate the degrees of freedom. Participant (slope and intercept) was considered a random effect, whereas age, sex, years of education, *APOE* ε4 status, and the rates of change in the above-mentioned neuroimaging measures and eTIV were considered fixed effects. The rates of change were calculated by dividing the changes in the variables between baseline and follow-up by the follow-up duration (months). The results are presented as standardized β values with their corresponding 95% CIs to represent the strength of their association. Two-tailed *P-*values < 0.05 were considered statistically significant for all analyses. All statistical analyses were performed using the SAS version 9.4 software (SAS Institute, Inc., Cary, NC, USA) and SPSS version 29.0 software (SPSS Inc., Chicago, IL, USA).

## Results

**Fig. 1:**
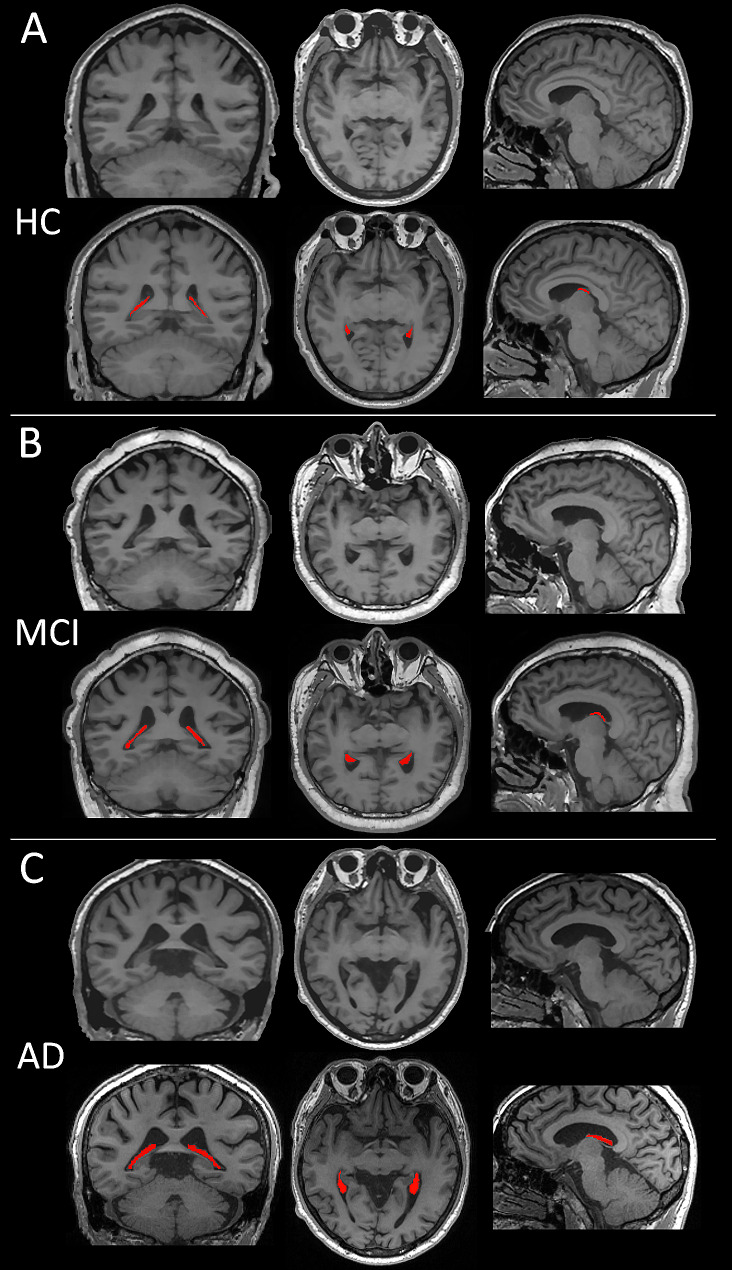
Comparisons among three representative 3D-T1 weighted images of the ChP volume in the three groups. The ChP volume (red) is the largest in patients with AD dementia, followed by those with MCI and HCs. All participants are 72-year-old men. The MMSE and MoCA scores of the HC are 30 and 28, respectively **(A)**. The MoCA score is 23 in patients with MCI with 10 years of education **(B)**, whereas the MoCA score is 17 in patients with AD with 13 years of education **(C)**

The analysis included 607 participants [age: 65.99 ± 8.79 years, females: 363 (59.80%); HCs, *n* = 110; MCI, *n* = 269; and AD dementia, *n* = 228]. Of the 497 patients on the AD continuum, 138 (27.77%) underwent lumbar puncture for CSF hallmark testing (MCI, *n* = 33; AD dementia, *n* = 105). Table 1 presents the participants’ demographic, clinical, CSF, and neuroimaging characteristics at baseline. Patients with AD dementia exhibited higher ChP volume and ChP/eTIV compared to that of patients with MCI and HCs (all *P* < 0.001). Figure 1 depicts representative 3D T1-weighted images of ChP volume in age- and sex-matched patients from the three groups.

**Table 1:** Demographic, clinical, CSF, and neuroimaging characteristics of all participants at baseline

Variables	HCs (*n* = 110)	MCI (*n* = 269)	AD (*n* = 228)	F/χ^2^/K	*P*_*T*_ value	*P*_*1*_ value	*P*_*2*_ value	*P*_*3*_ value
**Demographics (** ***N*** **=607)**								
Age [years, mean ± SD]	60.44 ± 7.13	64.83 ± 7.61	70.04 ± 8.96	57.62	< 0.001	< 0.001	< 0.001	< 0.001
Sex [female, *n* (%)]	62 (56.36)	168 (62.45)	133 (58.33)	1.53	0.465	0.271	0.731	0.349
BMI [kg/m^2^, mean ± SD]	23.79 ± 2.67	24.14 ± 3.27	23.57 ± 3.43	1.79	0.168	0.590	0.940	0.207
Education [years, median (IQR)]	16 (12, 16)	12 (9, 14)	9 (6, 12)	86.58	< 0.001	< 0.001	< 0.001	< 0.001
*APOE* ε4 status [yes, *n* (%)]	16 (14.55)	66 (24.54)	103 (45.17)	40.52	< 0.001	0.030	< 0.001	< 0.001
**Medical history (*N*** **= 607)**								
Hypertension [yes, *n* (%)]	36 (32.73)	113 (42.01)	103 (45.18)	4.78	0.091	0.093	0.029	0.478
Diabetes mellitus [yes, *n* (%)]	14 (12.73)	50 (18.59)	42 (18.42)	2.09	0.351	0.167	0.187	0.962
Stroke [yes, *n* (%)]	8 (7.27)	45 (16.73)	49 (21.49)	10.73	0.005	0.016	0.001	0.177
CHD [yes, *n* (%)]	13 (11.82)	47 (17.47)	50 (21.93)	3.94	0.139	0.108	0.047	0.570
Hyperlipidemia [yes, *n* (%)]	42 (38.18)	125 (46.47)	86 (37.72)	4.56	0.102	0.140	0.935	0.049
Smoking [yes, *n* (%)]	18 (16.36)	58 (21.56)	44 (19.30)	1.38	0.501	0.251	0.514	0.534
Alcohol consumption [yes, *n* (%)]	28 (25.45)	74 (27.51)	58 (25.44)	0.33	0.848	0.682	0.997	0.602
**CSF biomarkers (*N*** **= 138)**		MCI (n = 33)	AD (n = 105)					
Aβ_42_ [pg/L, median (IQR)]		0.61 (0.38, 0.75)	0.37 (0.26, 0.48)	-3.18	0.001			
Aβ_40_ [pg/L, median (IQR)]		12.36 (5.27, 9.76)	7.29 (5.27, 9.76)	-3.03	0.002			
Aβ_42/40_ [median (IQR)]		0.06 (0.04, 0.09)	0.05 (0.04, 0.07)	-0.27	0.791			
tTau [pg/L, median (IQR)]		0.49 (0.22, 0.74)	0.58 (0.38, 0.92)	-2.10	0.036			
pTau_181_ [ng/L, median (IQR)]		46.51 (34.60, 63.29)	53.80 (40.18, 98.56)	-2.45	0.014			
**Neuropsychological tests (*N*** **= 607)**								
MMSE [score, median (IQR)]	29 (28–30)	26 (24–28)	16 (9–22)	344.73	< 0.001	< 0.001	< 0.001	< 0.001
MoCA [score, median (IQR)]	26 (25–28)	22 (19–24)	11 (5–16)	394.19	< 0.001	< 0.001	< 0.001	< 0.001
NPI [score, median (IQR)]	0	1 (0–6)	13 (5–24)	282.33	< 0.001	< 0.001	< 0.001	< 0.001
ADL [score, median (IQR)]	20	20	30 (25–40)	483.91	< 0.001	1.000	< 0.001	< 0.001
**Neuroimaging volume measures (*N*** **= 607)**								
ChP [cm^3^, median (IQR)]	1.80 (1.47–2.19)	2.01 (1.77–2.38)	2.40 (2.07–2.97)	104.45	< 0.001	< 0.001	< 0.001	< 0.001
ChP/eTIV [×10^− 3^, median (IQR)]	1.21 (1.03–1.44)	1.53 (1.33–1.76)	1.82 (1.58–2.11)	161.28	< 0.001	< 0.001	< 0.001	< 0.001
eTIV [cm^3^, Mean ± SD]	1473.51 ± 157.47	1351.12 ± 154.15	1372.25 ± 199.93	19.95	< 0.001	< 0.001	< 0.001	0.528
Cortex [cm^3^, Mean ± SD]	433.72 ± 40.96	420.21 ± 42.75	381.83 ± 49.23	66.42	< 0.001	0.025	< 0.001	< 0.001
Subcortical GM [cm^3^, Mean ± SD]	55.84 ± 5.28	52.73 ± 5.17	47.82 ± 6.02	91.36	< 0.001	< 0.001	< 0.001	< 0.001
Hippocampus [cm^3^, median (IQR)]	8.69 (8.09–9.21)	7.96 (7.37–8.72)	6.47 (5.79–7.35)	215.45	< 0.001	< 0.001	< 0.001	< 0.001
LVV [cm^3^, median (IQR)]	18.09 (12.74–23.61)	20.51 (14.03–29.99)	37.72 (27.46–50.00)	177.37	< 0.001	0.014	< 0.001	< 0.001
WMH [cm^3^, median (IQR)]	1.08 (0.69–1.55)	1.79 (1.11–3.35)	4.81 (2.50–9.06)	207.67	< 0.001	< 0.001	< 0.001	< 0.001
ECT [cm, median (IQR)]	3.60 ± 0.27	3.30 ± 0.31	2.82 ± 0.47	185.22	< 0.001	< 0.001	< 0.001	< 0.001

*P*_*T*_ values < 0.05 were considered statistically significant. Statistical analyses were performed using the R × C diagram χ^²^ test, univariate analysis of variance, or Kruskal–Wallis test for all variables, except for CSF biomarkers, and the Mann–Whitney U test

*P*_*1*_, *P*_*2*_, *and P*_*3*_ values represent post hoc pairwise comparisons between patients with MCI and HCs, AD and HCs, AD and MCI, and were adjusted using Bonferroni correction (< 0.0125 indicated statistical significance)

Abbreviations: HCs, healthy controls; MCI, mild cognitive impairment; AD, Alzheimer’s disease; BMI, body mass index; *APOE* ε4, apolipoprotein E type epsilon 4; CHD, coronary heart disease; CSF, cerebrospinal fluid; Aβ, beta-amyloid; tTau, total Tau protein; pTau, phosphorylated Tau protein; MMSE, Mini-Mental State Examination; MoCA, Montreal Cognitive Assessment; NPI, Neuropsychiatric Inventory; ADL, Activities of Daily Living; ChP, choroid plexus; eTIV, estimated total intracranial volume; GM, gray matter; LVV, lateral ventricular volume; WMH, white matter hypointensity; ECT, entorhinal cortical thickness

### Baseline correlations of ChP with CSF hallmarks, neuropsychological tests, and multimodal neuroimaging measures in the AD continuum

**Fig. 2:**
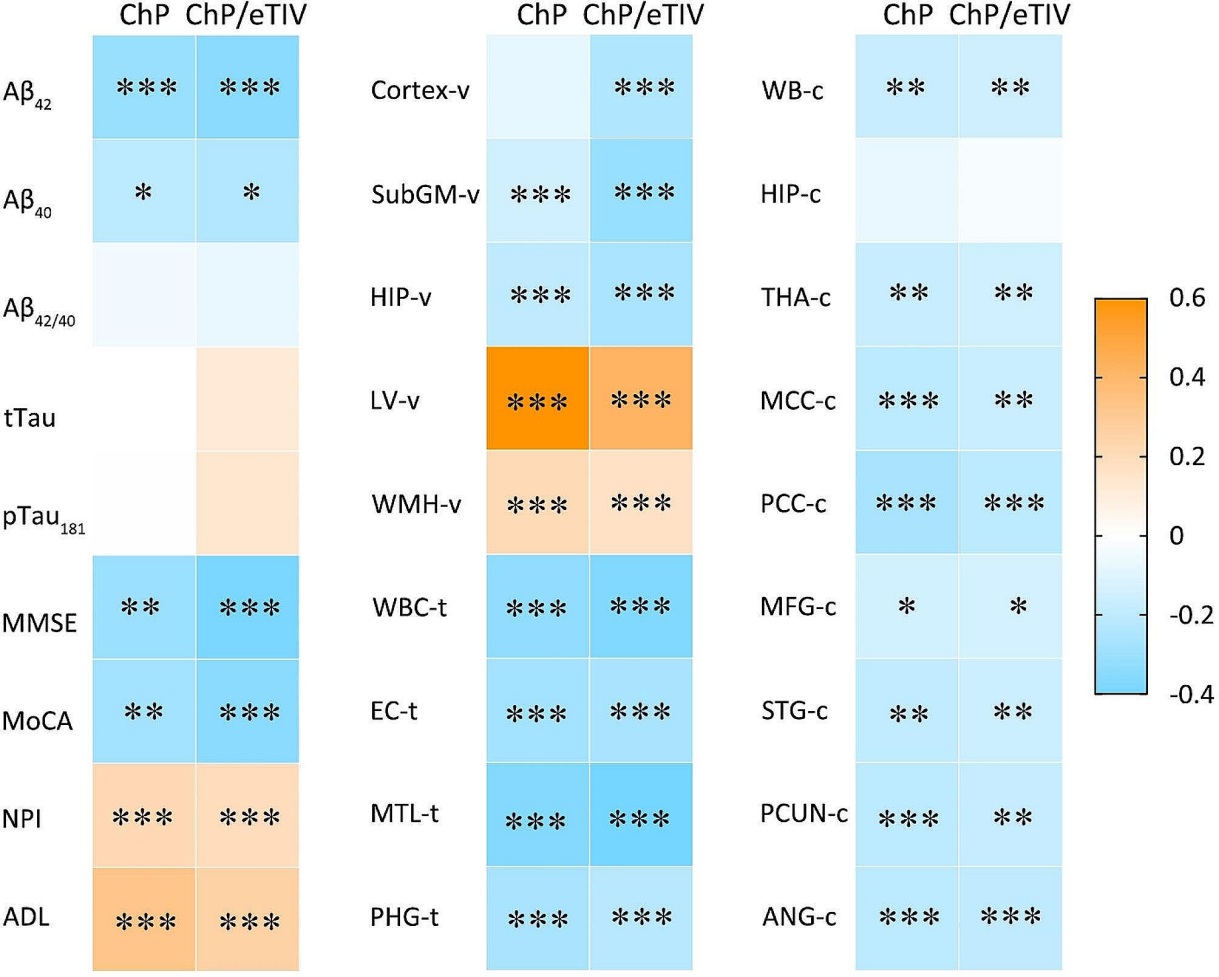
Heatmap of the baseline correlation between ChP volume (and ChP/eTIV) and CSF biomarkers, neuropsychological tests, and multimodal neuroimaging in the Alzheimer’s continuum. Partial Spearman’s correlation is performed after adjusting for age, sex, years of education, *APOE* ε4 status, and eTIV. -v, volume of the brain region; -t, cortical thickness of the brain region; -c, corrected cerebral blood flow (cCBF) of the brain region. The number of CSF analyses is 138, the number of regional cCBF is 288, and the number of neuropsychological assessments and brain structural measures is 497. The asterisks represent significant results: *P < 0.05; **P < 0.01; ***P < 0.001

Figure 2 illustrates a heatmap showing the correlation of ChP volume with CSF hallmarks, neuropsychological tests, and multimodal neuroimaging measures at baseline in patients on the AD continuum.

ChP volume (and ChP/eTIV) enlargement was correlated with decreased CSF Aβ_42_ (*r*= -0.310) and Aβ_40_ levels (*r*=-0.207), MMSE (*r*=-0.301) and MoCA scores (*r*=-0.281), but increased NPI (*r* = 0.208) and ADL scores (*r* = 0.311). This enlargement was correlated with a decline in the subcortical GM (*r*=-0.150) and hippocampal (*r*=-0.197) volumes; average CT of the whole brain (*r*=-0.333), entorhinal cortex (*r*=-0.280), middle temporal lobes (*r*=-0.366), and parahippocampal gyrus (*r*=-0.262); and cCBF values of the whole brain (*r*= -0.176), thalamus (*r*= -0.177), middle cingulate cortex (*r*=-0.20), posterior cingulate cortex (*r*=-0.263), middle frontal cortex (*r*=-0.14), superior temporal cortex (*r*=-0.194), precuneus (*r*=-0.207), and angular gyrus (*r*=-0.208), after adjusting for confounding factors (all *P* < 0.05).

### Mediating effect of ChP volume on the association between CSF hallmarks and neuropsychological tests on the AD continuum

**Fig. 3:**
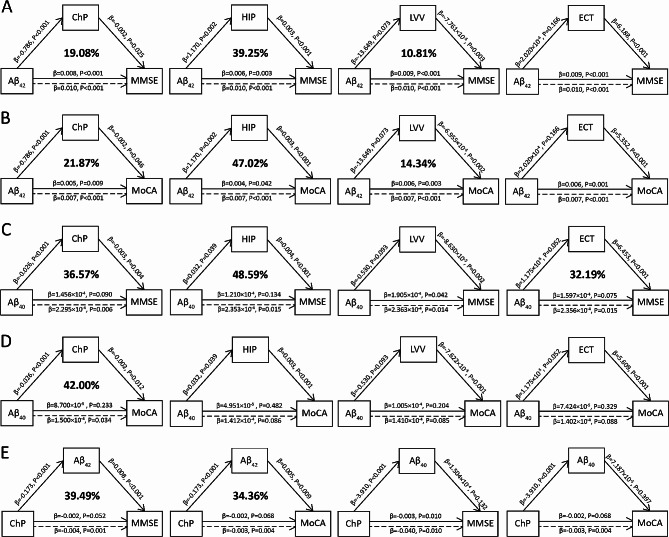
Simple mediating effects of ChP volume, hippocampal volume, LVV, and ECT alone on the association of CSF hallmarks and neuropsychological tests

Figure 3 presents the simple mediating effect of ChP volume, hippocampal volume, LVV, or ECT alone on the association between CSF biomarkers and neuropsychological tests on the AD continuum.

The associations between CSF Aβ_42_ levels and MMSE scores mediated by the ChP volume, hippocampal volume, and LVV alone were 19.08%, 39.25%, and 10.81%, respectively (Fig. 3A). Furthermore, the associations between CSF Aβ_42_ levels and MoCA scores mediated by the ChP volume, hippocampal volume, and LVV alone were 21.87%, 47.02%, and 14.34%, respectively (Fig. 3B). There was no mediating effect of ECT on these associations (all *P* > 0.05).

The associations between CSF Aβ_40_ levels and MMSE scores mediated by ChP volume, hippocampal volume, and ECT alone were 36.57%, 48.59%, and 32.19%, respectively (Fig. 3C). The associations between CSF Aβ_40_ levels and MoCA scores mediated by ChP volume was 42.00%; no mediating effect of hippocampal volume, LVV, or ECT was observed on the association between CSF Aβ_40_ levels and MoCA scores (all *P* > 0.05; Fig. 3D).

Notably, while the associations between ChP volume and MMSE and MoCA scores mediated by the CSF Aβ_42_ levels were 39.49% and 34.36%, respectively, no mediating effect of CSF Aβ_40_ levels on the association between ChP volume and these two scores was observed. All four neuroimaging measures exerted no mediating effect on the association between CSF hallmarks and the NPI score or ADL score (all *P* > 0.05).

### Diagnostic accuracy of ChP volume to identify cerebral pathological deposition and disease stages

**Fig. 4:**
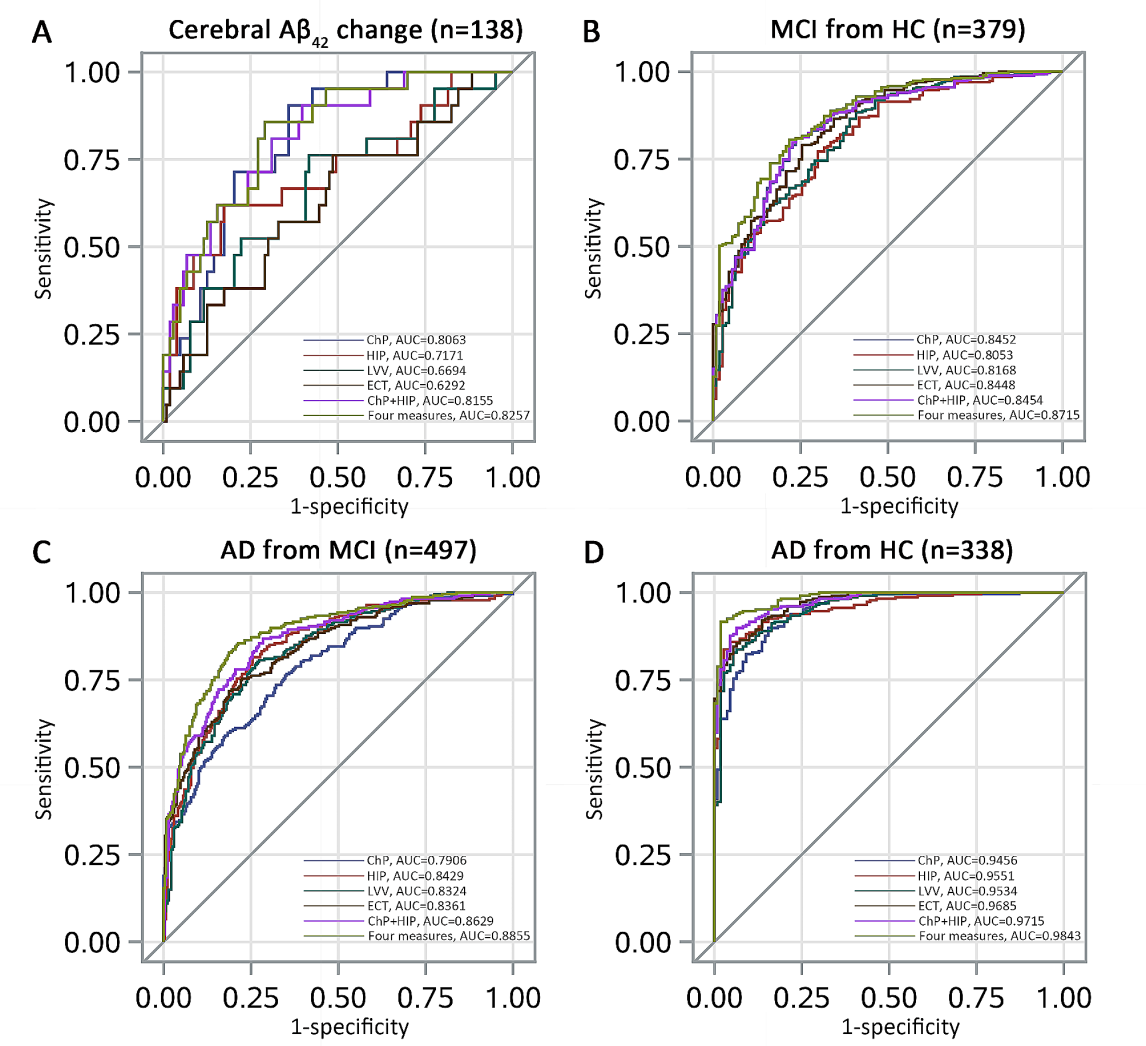
Receiver operating characteristic curves of four neuroimaging measures for discriminating the cerebral Aβ_42_ change and distinguishing different stages of the AD continuum. **(A)** The AUC of ChP volume alone is higher than that of LVV (*P* = 0.038) and ECT (*P* = 0.008) alone, and that of combined ChP and hippocampal volume is higher than that of hippocampal volume alone (*P* = 0.033). However, the combination of four neuroimaging measures (*P* = 0.443) or that of ChP and hippocampal volume (*P* = 0.735) does not show significant advantage over ChP volume alone; **(B)** The AUC of ChP volume alone is higher than those of hippocampal volume (*P* = 0.009) and LVV (*P* = 0.031) alone. The AUC of combined ChP and hippocampal volumes is higher than that of hippocampal volume alone (*P* = 0.009) but not higher than that of ChP volume alone (*P* = 0.654); (C) The AUC of combined ChP and hippocampal volumes is higher than those of hippocampal volume alone (*P* = 0.008) and ChP volume alone(*P* < 0.001); (D) The AUC of combined ChP and hippocampal volumes is higher than those of hippocampal volume alone (*P* = 0.004) and ChP volume alone (*P* = 0.001)

Figure 4 presents the ROC curves of four neuroimaging measures to identify the presence/absence of cerebral Aβ deposition and to differentiate among patients with AD dementia, MCI, and HCs.

ChP volume alone exhibited higher diagnostic accuracy in identifying cerebral Aβ_42_ changes on the AD continuum than LVV (AUC = 0.806 vs. 0.669, *P* = 0.038) and ECT alone (AUC = 0.806 vs.0.629, *P* = 0.008). No significant difference was observed between the ChP and hippocampal volume alone (AUC = 0.806 vs. 0.717, *P* = 0.166); however, the ability of combined ChP and hippocampal volume (AUC = 0.815 vs. 0.806, *P* = 0.735) or combined four neuroimaging measures (AUC = 0.826 vs. 0.806, *P* = 0.443) to identify cerebral Aβ_42_ changes showed no significant advantage compared with that of ChP volume alone (Fig. 4A).

ChP volume alone demonstrated higher diagnostic accuracy in differentiating patients with MCI from the HCs than hippocampal volume (AUC = 0.845 vs. 0.805, *P* = 0.009) and LVV alone (AUC = 0.845 vs.0.817, *P* = 0.031), although no significant difference was observed between ChP volume and EC thickness alone (AUC = 0.845 vs. 0.845, *P* = 0.981). However, combined ChP and hippocampal volume did not provide a significant advantage over ChP volume alone in differentiating patients with MCI from HCs (AUC = 0.845 vs. 0.845, *P* = 0.654; Fig. 4B).

ChP volume alone exhibited lower diagnostic accuracy in differentiating patients with AD from those with MCI than hippocampal volume (AUC = 0.791 vs. 0.843, *P* = 0.007), LVV (AUC = 0.791 vs. 0.832, *P* = 0.004), and ECT alone (AUC = 0.791 vs. 0.836, *P* = 0.018; Fig. 4C).

There was no significant difference regarding their ability to differentiate patients with AD dementia from HCs for ChP volume (AUC = 0.946), hippocampal volume (AUC = 0.955, *P* = 0.398), LVV (AUC = 0.953, *P* = 0.355) and ECT alone (AUC = 0.969, *P* = 0.05; Fig. 4D).

More importantly, when the ChP and hippocampal volumes were combined, the diagnostic efficiency for identifying cerebral Aβ_42_ changes and differentiating patients with AD dementia from those with MCI, patients with AD dementia from HCs, and patients with MCI from HCs increased to 0.816 (95%CI: 0.720–0.911), 0.863 (95%CI: 0.831–0.895), 0.972 (95%CI: 0.957–0.986), and 0.845 (95%CI: 0.803–0.888), respectively, all of which were significantly higher than the diagnostic efficiency of hippocampal volume alone (*P* = 0.029, *P* = 0.008, *P* = 0.004, and *P* = 0.009, respectively).

### Baseline and longitudinal associations between ChP volume and clinical presentations in patients across the AD continuum

Table 2 summarizes the association of ChP volume, hippocampal volume, LVV, ECT alone, and combined ChP and hippocampal volumes with the neuropsychological tests in patients across the AD continuum at baseline.

**Table 2:** Associations of ChP volume, hippocampal volume, LVV, ECT alone, and combined ChP and hippocampal volumes with the neuropsychological tests in patients in the AD continuum at baseline

Imaging markers	Outcomes	β value*	95% CI	t value	*P* value	Outcomes	β value*	95% CI	t value	*P* value
Model 1: only included one neuroimaging measure										
ChP alone	MMSE score	-0.32	-5.25 × 10^− 3^ to -2.99 × 10^− 3^	-7.15	< 0.001	NPI score	0.23	3.28 × 10^− 3^ to 8.84 × 10^− 3^	4.51	< 0.001
HIP alone		0.45	2.02 × 10^− 3^ to 3.01 × 10^− 3^	10.02	< 0.001		-0.19	-3.56 × 10^− 3^ to -1.10 × 10^− 3^	-3.73	< 0.001
LVV alone		-0.52	-2.51 × 10^− 4^ to -1.81 × 10^− 4^	-12.05	< 0.001		0.32	1.97 × 10^− 4^ to 3.75 × 10^− 4^	6.34	< 0.001
ECT alone		0.46	6.29 to 8.89	11.48	< 0.001		-0.24	-11.60 to -5.05	-4.99	< 0.001
ChP alone	MoCA score	-0.34	-5.31 × 10^− 3^ to -3.15 × 10^− 3^	-7.70	< 0.001	ADL score	0.31	4.08 × 10^− 3^ to 7.58 × 10^− 3^	6.53	< 0.001
HIP alone		0.47	2.16 × 10^− 3^ to 3.09 × 10^− 3^	11.09	< 0.001		-0.40	-4.08 × 10^− 3^ to -2.52 × 10^− 3^	-8.32	< 0.001
LVV alone		-0.52	-2.43 × 10^− 4^ to -1.76 × 10^− 4^	-12.23	< 0.001		0.58	2.99 × 10^− 4^ to 4.05 × 10^− 4^	13.03	< 0.001
ECT alone		0.45	6.13 to 8.62	11.67	< 0.001		-0.44	-12.90 to -8.83	-10.5	< 0.001
Model 2: both of ChP and hippocampal volume together										
ChP	MMSE score	-0.23	-4.05 × 10^− 3^ to -1.88 × 10^− 3^	-5.38	< 0.001	NPI score	0.19	2.49 × 10^− 3^ to 7.98 × 10^− 3^	3.75	< 0.001
HIP		0.39	1.70 × 10^− 3^ to 2.68 × 10^− 3^	8.73	< 0.001		-0.15	-3.01 × 10^− 3^ to -5.17 × 10^− 4^	-2.78	0.006
ChP	MoCA score	-0.24	-4.04 × 10^− 3^ to -2.01 × 10^− 3^	-5.84	< 0.001	ADL score	0.23	2.63 × 10^− 3^ to 6.07 × 10^− 3^	4.96	< 0.001
HIP		0.41	1.83 × 10^− 3^ to 2.76 × 10^− 3^	9.73	< 0.001		-0.34	-3.61 × 10^− 3^ to -2.04 × 10^− 3^	-7.09	< 0.001
Model 3: both of ChP, HIP, and CHP*HIP together										
ChP	MMSE score	-0.25	-0.01 to 0.01	-1.26	0.208	NPI score	0.15	-0.01 to 0.02	0.66	0.508
HIP		0.38	4.98 × 10^− 4^ to 3.76 × 10^− 3^	2.57	0.011		-0.17	-6.22 × 10^− 3^ to 2.02 × 10^− 3^	-1.00	0.318
ChP*HIP		0.02	6.39 × 10^− 7^ to 6.92 × 10^− 7^	0.08	0.002		0.04	1.54 × 10^− 6^ to 1.82 × 10^− 6^	0.17	0.867
ChP	MoCA score	0.12	-6.07 × 10^− 3^ to 3.16 × 10^− 3^	0.62	0.536	ADL score	0.31	-0.01 to 0.14	1.45	0.147
HIP		0.51	1.27 × 10^− 3^ to 4.33 × 10^− 3^	3.60	< 0.001		-0.28	-4.95 × 10^− 3^ to 2.24 × 10^− 4^	1.79	0.073
ChP*HIP		-0.15	8.41 × 10^− 7^ to 4.06 × 10^− 7^	0.69	0.493		-0.09	-1.25 × 10^− 6^ to 8.57 × 10^− 7^	-0.37	0.713

*N* = 497, adjusting for age, sex, years of education, *APOE* ε4 status, and eTIV. Abbreviations: AD, Alzheimer’s disease; MMSE, Mini-Mental State Examination; MoCA, Montreal Cognitive Assessment; NPI, Neuropsychiatric Inventory; ADL, Activities of Daily Living; ChP, choroid plexus volume; HIP, hippocampal volume; LVV, lateral ventricular volume; ECT, entorhinal cortical thickness; eTIV, estimated total intracranial volume

After correcting for age, sex, years of education, *APOE* ε4 status, and eTIV, ChP volume alone was associated with MMSE (β=-0.32, *P* < 0.001), MoCA (β=-0.34, *P* < 0.001), NPI (β = 0.23, *P* < 0.001), and ADL scores (β = 0.31, *P* < 0.001) at baseline (Table 2, Model 1). After correcting for hippocampal volume and the above-mentioned confounders, ChP volume was also associated with MMSE (β=-0.23, *P* < 0.001), MoCA (β=-0.24, *P* < 0.001), NPI (β = 0.19, *P* < 0.001), and ADL scores (β = 0.23, *P* = 0.006) at baseline (Table 2, Model 2). The interaction between ChP and hippocampal volumes was only observed for the MMSE score at baseline (*P* = 0.002; Table 2, Model 3).

Among the 497 patients on the AD continuum at baseline, 155 (MCI: 86 and AD: 69) were followed-up for an average of 10.0 ± 4.5 months. The clinical profiles of participants lost to follow-up, and those who completed follow-up are listed in Supplementary eTable 1. Table 3 presents the longitudinal associations between the rates of change in ChP volume, hippocampal volume, LVV, ECT alone, or combined ChP and hippocampal volumes and the rates of change in neuropsychological tests in patients in the AD continuum during the follow-up period.

**Table 3:** The longitudinal association of the rate of change in both ChP volume, hippocampal volume, LVV, ECT alone, or combined ChP and hippocampal volumes with the rate of changes in neuropsychological tests in patients on the AD continuum over follow-up

Imaging markers	Outcomes	β value*	95% CI	F value	*P* value	Outcomes	β value*	95% CI	F value	*P* value
Model 1: only included one neuroimaging measure										
ChP alone	MMSE rate	-0.19	-0.99–0.62	0.21	0.648	NPI rate	5.16	0.68–9.63	5.18	0.024
HIP alone		1.24	0.41–2.07	8.54	0.004		-0.75	-5.61–4.11	0.09	0.761
LVV alone		-0.17	-0.95–0.61	0.18	0.671		0.47	-3.93–4.87	0.04	0.833
ECT alone		1.18	0.40–1.96	8.94	< 0.001		-4.55	-17.14–8.04	0.51	0.476
ChP alone	MoCA rate	-0.18	-1.04–0.68	0.16	0.003	ADL rate	1.10	-0.29–2.48	2.45	0.120
HIP alone		0.09	-0.82–1.01	0.04	0.838		-0.42	-1.90–1.07	0.30	0.582
LVV alone		-0.36	-1.19–0.47	0.75	0.387		0.34	-1.01–1.69	0.25	0.621
ECT alone		0.46	-0.40–1.31	1.12	0.291		-0.48	-1.86–0.91	0.46	0.450
Model 2: both of ChP and hippocampal volume together										
ChP	MMSE rate	-0.09	-0.88–0.70	0.05	0.824	NPI rate	5.25	0.75–9.76	5.31	0.023
HIP		1.23	0.39–2.07	8.32	0.005		-1.22	-6.03–3.58	0.25	0.616
ChP	MoCA rate	-0.17	-1.04–0.70	0.15	0.699	ADL rate	1.07	-0.32–2.46	2.31	0.131
HIP		0.08	-0.84–1.00	0.03	0.865		-0.32	-1.81–1.17	0.18	0.672
Model 3: both of ChP, HIP, and CHP*HIP together										
ChP	MMSE rate	-0.11	-0.90–0.69	0.07	0.791	NPI rate	5.08	0.55–9.61	4.90	0.028
HIP		1.35	0.35–2.35	7.16	0.008		-2.42	-8.11–3.26	0.71	0.401
ChP*HIP		0.22	-0.73–1.16	0.21	0.651		-2.14	-7.52–3.25	0.61	0.435
ChP	MoCA rate	-0.21	-1.08–0.66	0.22	0.639	ADL rate	0.97	-0.42–2.37	1.90	0.171
HIP		0.34	-0.75–1.43	0.38	0.540		0.36	-1.39–2.11	0.17	0.684
ChP*HIP		0.46	-0.57–1.50	0.78	0.378		1.21	-0.45–2.87	2.08	0.152

*N* = 155, adjusting for age, sex, years of education, *APOE* ε4 status, and eTIV. Abbreviations: AD, Alzheimer’s disease; MMSE, Mini-Mental State Examination; MoCA, Montreal Cognitive Assessment; NPI, Neuropsychiatric Inventory; ADL, Activities of Daily Living; ChP, choroid plexus volume; HIP, hippocampal volume; LVV, lateral ventricular volume; ECT, entorhinal cortical thickness; eTIV, estimated total intracranial volume

After correcting for age, sex, years of education, *APOE* ε4 status, and the rate of change in eTIV, rapidly enlarged ChP volume was longitudinally associated with a faster increased in the NPI score (β = 5.16, *P* = 0.024; Table 3, Model 1). After correcting for hippocampal volume and the above-mentioned confounders, rapidly enlarged ChP volume was also longitudinally associated with a faster increased in the NPI score (β = 5.25, *P* = 0.023; Table 3, Model 2). Moreover, there was no interaction between ChP and hippocampal volumes in these associations (*P* = 0.435; Table 3, Model 3).

## Discussion

This study demonstrated that ChP volume enlargement was correlated with the CSF hallmarks of AD, neuropsychological changes, and multimodal neuroimaging measures across the AD continuum. Moreover, ChP volume mediated the association between CSF hallmarks (Aβ_42_ and Aβ_40_) and cognitive impairment. These findings preliminarily revealed that abnormalities in the ChP linked an upstream pathological event with downstream clinical manifestations and were closely related to multi-dimensional measures of neurodegenerative changes along the AD continuum. Moreover, we observed that ChP volume possessed higher diagnostic accuracy in identifying cerebral Aβ_42_ changes or differentiating patients with MCI from the HCs than hippocampal volume, LVV, or ECT alone after accounting for important demographic factors. Additionally, the combination of ChP and hippocampal volumes was more effective in identifying cerebral Aβ changes and discriminating between the presence of AD dementia, MCI, and normal cognition than hippocampal volume alone, suggesting the early diagnostic value of ChP volume on the AD continuum. Furthermore, we found that faster ChP volume enlargement was associated with more rapid deterioration in the severity of NPS during follow-up, which was not observed in the association of the hippocampal volume, LVV, or ECT with these clinical symptoms. Given the high heterogeneity of the AD continuum, our study provides evidence for a novel, reliable structural neuroimaging marker to assist in early detection and prediction of the AD continuum in clinical practice.

Consistent with our findings, a previous study also identified negative associations between ChP volume and CSF proteins (Aβ, total and phosphorylated-Tau) in patients with MCI or AD dementia [14]. To our knowledge, our study is the first to reveal that ChP volume possesses high diagnostic efficiency for identifying patients with MCI and HCs, which is higher than that of hippocampal volume, LVV, or ECT alone after accounting for important demographic factors. Moreover, when the ChP and hippocampal volumes were combined, the diagnostic efficiency for identifying the cerebral Aβ_42_ changes and differentiating patients with AD dementia from those with MCI, patients with AD dementia from HCs, and patients with MCI from HCs increased significantly compared with the diagnostic efficiency of hippocampal volume alone. Our findings suggest an advantageous assisted diagnostic value of ChP volume during the early stages of the AD continuum. The glymphatic system is a fluid-clearance pathway that promotes CSF/interstitial fluid exchange for effective waste–solute transport and drainage, including Aβ [35, 36]. In this process, the ChP plays a crucial role in regulating CSF dynamics and maintaining brain homeostasis [37]. Recent studies have indicated that Aβ accumulation and neurofibrillary tangle-like inclusions could lead to physiological dysfunction of the ChP, causing stromal fibrosis, stromal dystrophic calcification, blood vessel thickening, inflammation, and reduced CSF production, consequently affecting the structure and function of the ChP [38, 39]. Given that Aβ accumulation is a key pathological hallmark of the AD continuum, which is positively correlated with clinical progression and severity [40], we conducted mediation analyses and discovered that ChP volume mediated a robust association between CSF Aβ levels and cognitive impairment, and the CSF Aβ levels also mediated the association between ChP volume and cognitive impairment. Therefore, ChP abnormality could serve as a non-invasive surrogate marker of impaired Aβ clearance in the brain, which is crucial in connecting Aβ-related neurodegeneration with cognitive impairment in the AD continuum.

Consistently, we found a relationship between ChP volume enlargement and more severe atrophy and lower cerebral perfusion in well-known cognition-related brain regions, further confirming the underlying connection between ChP and these brain regions. Previous research exploring the association between ChP and other essential brain areas in the AD continuum is extremely scarce [19]. Recent studies indicated that ChP volume enlargement correlated with reduced cortical thickness and progressive local brain atrophy in patients with multiple sclerosis [41, 42]. ChP volume was significantly correlated with neuroinflammatory changes in the anterior cingulate and prefrontal cortex and circulating cytokine elevation in patients with depression [43, 44]. A murine model study revealed that impairment in the structure and transport function of the ChP was correlated with CSF nutritional composition alterations, which further affected hippocampal plasticity [45]. Similarly, another mouse injection experiment demonstrated that in vitro generated plasma extracellular vesicles (pEV) with similar characteristics as AD-pEV could accumulate in the ChP of injected animals and reach the primary hippocampal neurons [46], further indicating the connection among these brain regions. Collectively, these results solidified our hypothesis regarding a strong association between ChP volume and other neurodegenerative measures, thereby highlighting its significance in the pathophysiology of the AD continuum.

Consistent with our findings, retrospective studies also revealed that ChP volume was larger in patients with AD dementia than in those with MCI and HCs at baseline [14, 19]. Notably, we demonstrated that ChP volume serves as a potent novel measure for discriminating AD dementia, MCI, and normal cognition after accounting for important demographic factors, providing a unique insight into the clinical value of ChP in the AD continuum. A recent study found that ChP volume possessed high diagnostic efficiency in differentiating between patients with frontotemporal lobar degeneration and HCs [47], reiterating its pathological role in the neurodegenerative process. Importantly, we found that rapid ChP volume enlargement was longitudinally associated with accelerated deterioration in the severity of NPS during the follow-up period. The association was not merely a consequence of the decrease in whole brain or hippocampal volume involved in AD but represents an independent process with a higher predictive value than that of other well-known brain regions in the change of NPS. These findings suggest the early diagnostic and predictive value of ChP volume in the AD continuum. Although the exact mechanism regarding the association between the ChP volume and the NPS progression remains unclear, previous research indicated that ChP volume enlargement reflected aging and impaired ChP function, leading to blood–CSF barrier disruption and subsequent reduction in CSF Aβ clearance; in patients with AD, this disruption culminates in clinical progression and neurodegeneration [39, 48]. This result was also consistent with the above-mentioned findings and previous observations that ChP volume was strongly related to abnormal cerebral Aβ deposition, which triggers irreversible AD pathological processes [49, 50]. In addition, ChP plays a pivotal role in regulating the trafficking of immune cells from the brain parenchyma into the CSF. It has recently attracted attention as a key structure in the initiation of neuroinflammatory responses. The latter is another important pathophysiological hallmark of the onset and development of the AD continuum [51]. A recent retrospective study found that ChP volume was significantly positively associated with neuroinflammatory plasma biomarkers (GFAP) [52]. An observational study found that a significantly greater CP volume in patients with depression was positively correlated with [^11^C]PK11195 PET (neuro-inflammatory markers) binding in the anterior cingulate, prefrontal, and insular cortices, but not with the peripheral inflammatory markers [53]. A translational framework study also revealed that ChP enlargement was strongly linked to acute and ongoing neuroinflammatory activity in mouse models and in patients with multiple sclerosis. These findings suggest that the degradation of the ChP structure could co-occur with neuroinflammation, which could contribute to the development of neurodegeneration and clinical progression. However, a preliminary post-mortem study with in vivo findings revealed that the ChP damage in AD was consistent with increased cytokine levels but without evidence of inflammatory activation or infiltrates [16].

A notable strength of this study is its large-scale and longitudinal design, which facilitated in-depth exploration of the role and clinical values of ChP regarding the onset and development of neurodegeneration in the AD continuum through neuropsychological assessment for the first time. Nevertheless, this study had some limitations. First, the follow-up sample was relatively small owing to the loss of follow-up resulting from the COVID-19 pandemic. Nevertheless, it remains the largest longitudinal study investigating the role of the ChP in the AD continuum. Second, functional changes in the ChP were not identified using multimodal imaging (ASL and diffusion tensor imaging), but ChP volume provided simpler and more convenient and feasible structural imaging clinically. Finally, the invasive nature of lumbar puncture prevented analysis of the temporal dynamics between ChP volume and CSF biomarkers.

## Conclusions

This study reveals the overarching role of the ChP in neurodegeneration in the AD continuum by examining the relationships between ChP enlargement and increased cerebral Aβ accumulation, cognitive decline, and decreased cerebral volumes, cortical thickness, and perfusion, and the strong mediating effect of ChP volume on the association between upstream cerebral pathology and downstream clinical changes. It could better detect the early stages of the AD continuum and predict prognosis, and significantly enhance the differential diagnostic ability of hippocampus on the AD continuum. These findings indicate that ChP volume could be a non-invasive, sensitive, reliable, and translational imaging marker for early detection and prediction across the AD continuum. Future research should explore functional changes in the ChP, clinically validate this diagnostic and prognostic marker on the AD continuum, and determine whether clinical interventions targeting the ChP can improve cognitive function and outcomes.

### Electronic supplementary material

Below is the link to the electronic supplementary material.

Supplementary Material 1

## Data Availability

No datasets were generated or analysed during the current study.

## Declarations

### Abbreviations

Activities of Daily Living (ADL).

Alzheimer’s disease (AD).

Blood–brain barrier (BBB).

Body mass index (BMI).

Cerebrospinal fluid (CSF).

Cortical thickness (CT).

Choroid plexus (ChP).

Corrected cerebral blood flow (cCBF).

Chinese Imaging, Biomarkers, and Lifestyle (CIBL).

Healthy controls (HCs).

Mild cognitive impairment (MCI).

Mini-Mental State Examination (MMSE).

Montreal Cognitive Assessment (MoCA).

Neuropsychiatric Inventory (NPI).

Natural direct effects (NDEs).

Natural indirect effects (NIEs).

Receiver operating characteristic (ROC).

Gray matter (GM).

White matter hypointensity (WMH).

Total effect (TE).

## References

[1] Scheltens P, De Strooper B, Kivipelto M (2021). Alzheimer’s disease. Lancet.

[2] Kurz C, Walker L, Rauchmann BS (2022). Dysfunction of the blood-brain barrier in Alzheimer’s disease: evidence from human studies. Neuropathol Appl Neurobiol.

[3] Jack CR, Bennett DA, Blennow K (2018). NIA-AA Research Framework: toward a biological definition of Alzheimer’s disease. Alzheimers Dement.

[4] Jia J, Ning Y, Chen M (2024). Biomarker changes during 20 years preceding Alzheimer’s Disease. N Engl J Med.

[5] Kompaníková P, Bryja V (2022). Regulation of choroid plexus development and its functions. Cell Mol Life Sci.

[6] Čarna M, Onyango IG, Katina S (2023). Pathogenesis of Alzheimer’s disease: involvement of the choroid plexus. Alzheimers Dement.

[7] Ramusino MC, Vitali P, Anzalone N (2022). Vascular lesions and brain atrophy in Alzheimer’s, vascular and mixed dementia: an optimized 3T MRI protocol reveals distinctive radiological profiles. Curr Alzheimer Res.

[8] Tadayon E, Pascual-Leone A, Press D (2020). Choroid plexus volume is associated with levels of CSF proteins: relevance for Alzheimer’s and Parkinson’s disease. Neurobiol Aging.

[9] Alawode DOT, Heslegrave AJ, Ashton NJ (2021). Transitioning from cerebrospinal fluid to blood tests to facilitate diagnosis and disease monitoring in Alzheimer’s disease. J Intern Med.

[10] Chuluunbat M, Matsuda D, Fujita K (2022). Identification and validation of a gray matter volume network in Alzheimer’s disease. J Neurol Sci.

[11] Ossenkoppele R, Smith R, Ohlsson T (2019). Associations between tau, Aβ, and cortical thickness with cognition in Alzheimer disease. Neurology.

[12] Duan W, Sehrawat P, Balachandrasekaran A (2020). Cerebral blood Flow is Associated with Diagnostic Class and Cognitive decline in Alzheimer’s Disease. J Alzheimers Dis.

[13] Yu J, Li J, Huang X (2012). The Beijing version of the Montreal Cognitive Assessment as a brief screening tool for mild cognitive impairment: a community-based study. BMC Psychiatry.

[14] Leung VP, Lam LC, Chiu HF (2001). Validation study of the Chinese version of the neuropsychiatric inventory (CNPI). Int J Geriatr Psychiatry.

[15] Kecskemeti S, Freeman A, Travers BG (2021). FreeSurfer based cortical mapping and T1-relaxometry with MPnRAGE: test-retest reliability with and without retrospective motion correction. NeuroImage.

[16] Veitch DP, Weiner MW, Aisen PS (2019). Understanding disease progression and improving Alzheimer’s disease clinical trials: recent highlights from the Alzheimer’s Disease Neuroimaging Initiative. Alzheimers Dement.

[17] Lombardi G, Crescioli G, Cavedo E (2020). Structural magnetic resonance imaging for the early diagnosis of dementia due to Alzheimer’s disease in people with mild cognitive impairment. Cochrane Database Syst Rev.

[18] Wang X, Huang W, Su L (2020). Neuroimaging advances regarding subjective cognitive decline in preclinical Alzheimer’s disease. Mol Neurodegener.

[19] Saunders NR, Dziegielewska KM, Fame RM (2023). The choroid plexus: a missing link in our understanding of brain development and function. Physiol Rev.

[20] Kant S, Stopa EG, Johanson CE (2018). Choroid plexus genes for CSF production and brain homeostasis are altered in Alzheimer’s disease. Fluids Barriers CNS.

[21] Scarpetta V, Bodaleo F, Salio C (2023). Morphological and mitochondrial changes in murine choroid plexus epithelial cells during healthy aging. Fluids Barriers CNS.

[22] Gião T, Teixeira T, Almeida MR (2022). Choroid Plexus in Alzheimer’s Disease-The Current State of Knowledge. Biomedicines.

[23] Choi JD, Moon Y, Kim HJ (2022). Choroid Plexus volume and permeability at Brain MRI within the Alzheimer Disease Clinical Spectrum. Radiology.

[24] Jiang J, Hong Y, Li W (2023). Chain Mediation Analysis of the Effects of Nutrition and Cognition on the Association of Apolipoprotein E ɛ4 with neuropsychiatric symptoms in Alzheimer’s Disease. J Alzheimers Dis.

[25] Jiang J, Liu Y, Wang A (2023). Development and validation of a nutrition-related genetic-clinical-radiological nomogram associated with behavioral and psychological symptoms in Alzheimer’s disease. Chin Med J (Engl).

[26] McKhann GM, Knopman DS, Chertkow H (2011). The diagnosis of dementia due to Alzheimer’s disease: recommendations from the National Institute on Aging-Alzheimer’s Association workgroups on diagnostic guidelines for Alzheimer’s disease. Alzheimers Dement.

[27] Katzman R, Zhang MY, Ouang-Ya-Qu (1988). A Chinese version of the Mini-mental State examination; impact of illiteracy in a Shanghai dementia survey. J Clin Epidemiol.

[28] Leuzy A, Mattsson-Carlgren N, Palmqvist S (2022). Blood-based biomarkers for Alzheimer’s disease. EMBO Mol Med.

[29] Márquez F, Yassa MA (2019). Neuroimaging biomarkers for Alzheimer’s Disease. Mol Neurodegener.

[30] Chandra A, Dervenoulas G, Politis M, Alzheimer’s Disease Neuroimaging Initiative (2019). Magnetic resonance imaging in Alzheimer’s disease and mild cognitive impairment. J Neurol.

[31] Isensee F, Jaeger PF, Kohl SAA (2021). nnU-Net: a self-configuring method for deep learning-based biomedical image segmentation. Nat Methods.

[32] Monereo-Sánchez J, de Jong JJA, Drenthen GS (2021). Quality control strategies for brain MRI segmentation and parcellation: practical approaches and recommendations - insights from the Maastricht study. NeuroImage.

[33] Muller J, Sinnecker T, Wendebourg MJ (2022). Choroid Plexus volume in multiple sclerosis vs Neuromyelitis Optica Spectrum disorder: a Retrospective, cross-sectional analysis. Neurol Neuroimmunol Neuroinflamm.

[34] Serang S, Jacobucci R (2020). Exploratory Mediation Analysis of Dichotomous Outcomes via regularization. Multivar Behav Res.

[35] Lohela TJ, Lilius TO, Nedergaard M (2022). The glymphatic system: implications for drugs for central nervous system diseases. Nat Rev Drug Discov.

[36] Hablitz LM, Nedergaard M (2021). The Glymphatic System: a Novel Component of Fundamental Neurobiology. J Neurosci.

[37] Christensen J, Li C, Mychasiuk R (2022). Choroid plexus function in neurological homeostasis and disorders: the awakening of the circadian clocks and orexins. J Cereb Blood Flow Metab.

[38] Shen X, Xia L, Liu L (2020). Altered clearance of beta-amyloid from the cerebrospinal fluid following subchronic lead exposure in rats: roles of RAGE and LRP1 in the choroid plexus. J Trace Elem Med Biol.

[39] Municio C, Carrero L, Antequera D (2023). Choroid Plexus aquaporins in CSF Homeostasis and the Glymphatic System: their relevance for Alzheimer’s Disease. Int J Mol Sci.

[40] Hampel H, Hardy J, Blennow K (2021). The Amyloid-β pathway in Alzheimer’s Disease. Mol Psychiatry.

[41] Chen X, Luo D, Zheng Q (2023). Enlarged choroid plexus related to cortical atrophy in multiple sclerosis. Eur Radiol.

[42] Ricigliano VAG, Morena E, Colombi A (2021). Choroid Plexus Enlargement in Inflammatory multiple sclerosis: 3.0-T MRI and translocator protein PET evaluation. Radiology.

[43] Bravi B, Melloni EMT, Paolini M (2023). Choroid plexus volume is increased in mood disorders and associates with circulating inflammatory cytokines. Brain Behav Immun.

[44] Althubaity N, Schubert J, Martins D (2022). Choroid plexus enlargement is associated with neuroinflammation and reduction of blood brain barrier permeability in depression. Neuroimage Clin.

[45] Liu K, Li H, Zeng N (2023). Decline of stress resilience in aging rats: focus on choroid plexus-cerebrospinal fluid-hippocampus. World J Biol Psychiatry.

[46] Lee JH, Ostalecki C, Oberstein T (2022). Alzheimer’s disease protease-containing plasma extracellular vesicles transfer to the hippocampus via the choroid plexus. EBioMedicine.

[47] Assogna M, Premi E, Gazzina S (2023). Association of Choroid Plexus volume with serum biomarkers, clinical features, and Disease Severity in patients with Frontotemporal Lobar Degeneration Spectrum. Neurology.

[48] Novakova Martinkova J, Ferretti MT, Ferrari A (2023). Longitudinal progression of choroid plexus enlargement is associated with female sex, cognitive decline and ApoE E4 homozygote status. Front Psychiatry.

[49] Palmqvist S, Insel PS, Stomrud E (2019). Cerebrospinal fluid and plasma biomarker trajectories with increasing amyloid deposition in Alzheimer’s disease. EMBO Mol Med.

[50] Lee WJ, Brown JA, Kim HR (2022). Regional Aβ-tau interactions promote onset and acceleration of Alzheimer’s disease tau spreading. Neuron.

[51] Lee H, Ozturk B, Stringer MS (2022). Choroid plexus tissue perfusion and blood to CSF barrier function in rats measured with continuous arterial spin labeling. NeuroImage.

[52] Bouhrara M, Walker KA, Alisch R. Association of Plasma Markers of Alzheimer’s Disease, Neurodegeneration, and Neuroinflammation with the Choroid Plexus Integrity in Aging. Aging Dis. 2024 Jan;8. 10.14336/AD.2023.1226. 10.14336/AD.2023.1226 PMC11346414 38300640

[53] Fleischer V, Gonzalez-Escamilla G, Ciolac D (2021). Translational value of choroid plexus imaging for tracking neuroinflammation in mice and humans. Proc Natl Acad Sci U S A.

