# Supplementary material for: Choroid plexus volume as a novel candidate neuroimaging marker of the Alzheimer’s continuum
Source: Alzheimers Res Ther. 2024 Jul 3;16:149. doi: 10.1186/s13195-024-01520-w (PMC11221040; doi:10.1186/s13195-024-01520-w)
Supplement: Supplementary file 1 — Supplementary Material 1 [file 13195_2024_1520_MOESM1_ESM.docx]

# Choroid Plexus Volume: A Novel Candidate Neuroimaging Marker of the Alzheimer’s Continuum

eFigure 1. A detailed flowchart of the eligibility criteria at baseline and follow-up………………….2

S01. STROBE Statement.…………………………………………………………………………………………………….……3

S02. The detailed comprehensive neuropsychological assessments………………………………….…….5

S03. The detailed parameters and processing of multimodal neuroimaging………………….………..6

Structural 3D-T1 weighted imaging………………………………………………………………………….………6

7-delay pCASL………………………………………………………………………………………………………….………6

eTable S1. Baseline clinical profiles of patients with or without follow-up……………………………….7

References ……………………………………………………………………………………………………………………………..8

# eFigure 1 A detailed flowchart of the eligibility criteria at baseline and follow-up


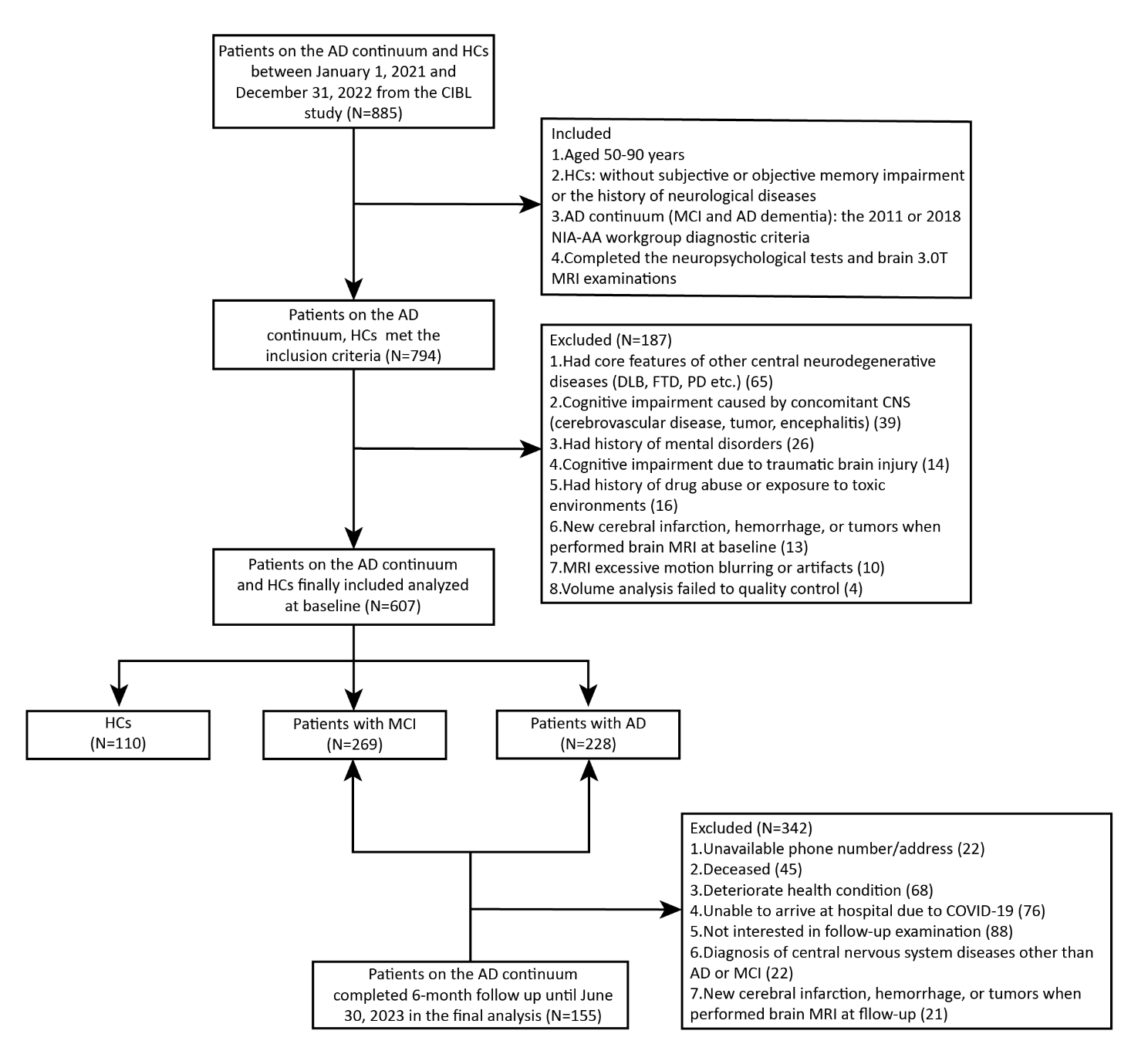


Abbreviations: MCI, mild cognitive impairment; AD, Alzheimer’s disease; HCs, healthy controls; CIBL, Chinese Imaging, Biomarkers, and Lifestyle Study; NIA-AA, National Institute on Aging–Alzheimer’s Association; DLB, dementia with Lewy bodies; FTD, frontotemporal dementia; PD, Parkinson’s disease; CNS, central nervous system; MRI, magnetic resonance imaging; COVID-19, coronavirus disease 2019

# S01. STROBE Statement—Checklist of items that should be included in reports of cohort studies

|  | Item No | Recommendation | Page No |
| --- | --- | --- | --- |
| **Title and abstract** | 1 | (*a*) Indicate the study’s design with a commonly used term in the title or the abstract | 1-3 |
|  |  | (*b*) Provide in the abstract an informative and balanced summary of what was done and what was found | 2-3 |
| Introduction | | | |
| Background/rationale | 2 | Explain the scientific background and rationale for the investigation being reported | 4-5 |
| Objectives | 3 | State specific objectives, including any prespecified hypotheses | 5-6 |
| Methods | | | |
| Study design | 4 | Present key elements of study design early in the paper | 6 |
| Setting | 5 | Describe the setting, locations, and relevant dates, including periods of recruitment, exposure, follow-up, and data collection | 6 |
| Participants | 6 | (*a*) Give the eligibility criteria, and the sources and methods of selection of participants. Describe methods of follow-up | 6, suppl. 2-3 |
|  |  | (*b*) For matched studies, give matching criteria and number of exposed and unexposed |  |
| Variables | 7 | Clearly define all outcomes, exposures, predictors, potential confounders, and effect modifiers. Give diagnostic criteria, if applicable | 6-11 |
| Data sources/ measurement | 8* | For each variable of interest, give sources of data and details of methods of assessment (measurement). Describe comparability of assessment methods if there is more than one group | 6-11 |
| Bias | 9 | Describe any efforts to address potential sources of bias | 8-11 |
| Study size | 10 | Explain how the study size was arrived at | 6 |
| Quantitative variables | 11 | Explain how quantitative variables were handled in the analyses. If applicable, describe which groupings were chosen and why | 8-11 |
| Statistical methods | 12 | (*a*) Describe all statistical methods, including those used to control for confounding | 8-11 |
|  |  | (*b*) Describe any methods used to examine subgroups and interactions | 8-11 |
|  |  | (*c*) Explain how missing data were addressed | 8-11 |
|  |  | (*d*) If applicable, explain how loss to follow-up was addressed | 8-11 |
|  |  | (*e*) Describe any sensitivity analyses | 8-11 |
| Results | | |  |
| Participants | 13* | (a) Report numbers of individuals at each stage of study—eg numbers potentially eligible, examined for eligibility, confirmed eligible, included in the study, completing follow-up, and analysed | 11 |
|  |  | (b) Give reasons for non-participation at each stage | eFigure 1 |
|  |  | (c) Consider use of a flow diagram | eFigure 1 |
| Descriptive data | 14* | (a) Give characteristics of study participants (eg demographic, clinical, social) and information on exposures and potential confounders | 11 Table 1 |
|  |  | (b) Indicate number of participants with missing data for each variable of interest | 11-14, eFigure 1 |
|  |  | (c) Summarise follow-up time (eg, average and total amount) | 14, eTable 1 |
| Outcome data | 15* | Report numbers of outcome events or summary measures over time | 14 |
| Main results | 16 | (*a*) Give unadjusted estimates and, if applicable, confounder-adjusted estimates and their precision (eg, 95% confidence interval). Make clear which confounders were adjusted for and why they were included | 8-14 |
|  |  | (*b*) Report category boundaries when continuous variables were categorized | 11-14 |
|  |  | (*c*) If relevant, consider translating estimates of relative risk into absolute risk for a meaningful time period | Not suitable |
| Other analyses | 17 | Report other analyses done—eg analyses of subgroups and interactions, and sensitivity analyses | 11-14 |
| Discussion | | | |
| Key results | 18 | Summarise key results with reference to study objectives | 15 |
| Limitations | 19 | Discuss limitations of the study, taking into account sources of potential bias or imprecision. Discuss both direction and magnitude of any potential bias | 19 |
| Interpretation | 20 | Give a cautious overall interpretation of results considering objectives, limitations, multiplicity of analyses, results from similar studies, and other relevant evidence | 15-19 |
| Generalisability | 21 | Discuss the generalisability (external validity) of the study results | 19 |
| Other information | | | |
| Funding | 22 | Give the source of funding and the role of the funders for the present study and, if applicable, for the original study on which the present article is based | 22 |

*Give information separately for exposed and unexposed groups.

**Note:** An Explanation and Elaboration article discusses each checklist item and gives methodological background and published examples of transparent reporting. The STROBE checklist is best used in conjunction with this article (freely available on the Web sites of PLoS Medicine at http://www.plosmedicine.org/, Annals of Internal Medicine at http://www.annals.org/, and Epidemiology at http://www.epidem.com/). Information on the STROBE Initiative is available at <http://www.strobe-statement.org>.

# S02. Detailed comprehensive neuropsychological assessments

Global cognition was evaluated using the Chinese version of the MMSE [1] and Beijing version of the MoCA [2]. Participants with a total MMSE score of ≤ 24 for > 6 y of education, ≤ 20 for 1–6 y of education, and ≤ 17 for 0 y of education, or a total MoCA score of ≤ 24 for > 6 years of education, ≤ 20 for 1–6 years of education, and ≤ 14 for 0 years of education were considered indicative of cognitive impairment.

The NPI is a structured interview of caregivers to evaluate 12 different behavioral and neuropsychiatric domains, including delusions, hallucinations, agitation, depression, anxiety, euphoria, apathy, disinhibition, irritability, aberrant motor activity, sleep disturbances, and appetite disturbances [3]. The NPI scale grades each reported symptom using a severity score (rated on a 3-point scale) and a frequency score (rated on a 4-point scale). The total NPI severity scores were calculated by adding up the 12 severity sub-scores (severity score × frequency score).

The ADL questionnaire was used to evaluate the patient’s daily function, including 10 basic ADL tasks and 10 instrumental ADL tasks. The total score (range: 20–80) was the sum of individual scores for each item rated on a 4-point scale.

# S03. The detailed parameters and processing of multimodal neuroimaging

# Structural 3D-T1-wighted imaging

# High-resolution 3D T1 scans were performed using the inversion recovery gradient recalled echo sequence with the following parameters: repetition time (TR)=7.3 ms, echo time (TE)=3.0 ms, inversion time=450 ms; flip angle=12°, field of view (FOV)=256 mm×256 mm, acquisition matrix=256×256, slice thickness=1.0 mm, slice number=176, and scan time=4 min 56 s. For each participant, lesions on the T1 images were filed using the default lesion segmentation tool pipeline. After lesion-filing, the 3D T1-weighted images were segmented.

# 7-delay pCASL

Scan parameters of the time-encoded 7-delay pCASL sequence to obtain the CBF values were: TR = 9315.0 ms, TE = 11.2 ms, FOV = 220 mm × 220 mm, acquisition matrix = 512 × 512, 48 axial slices, thickness = 3.0 mm; the label durations of the seven labeling blocks were 0.361, 0.378, 0.402, 0.436, 0.491, 0.591, and 0.842 s, the post-labeling delays were 1.000, 1.361, 1.739, 2.141, 2.577, 3.067, and 3.658 s, and the scan time was 15 min 55 s. A junior radiologist with 5 years of experience in neuroradiology performed data processing using CereFlow software 1.0 (Anying Technology Beijing Co., Ltd., China), which was checked by a senior radiologist with 20 years of experience in neuroradiology. The following steps were performed: (1) importing automatically generated cerebral blood flow (CBF) and arterial transit time (ATT) images from the default vendor’s postprocessing pipeline; (2) co-registration of the M0 image (GE ASL’s PD image) with the anatomical T1-weighted image; the CBF/ATT images were also co-registered to the T1 image with the same transformation parameters; (3) normalization of the T1 images to the Montreal Neurological Institute template; (4) warping the CBF/ATT images into the Montreal Neurological Institute space using the forward transformation matrix derived from T1; and (5) regional CBF corrected by ATT (cCBF) were reconstructed.

**eTable 1. The baseline clinical profiles of patients with follow-up and those lost to follow-up**

| Variable | Overall  (N=497) | Patients followed up  (N=155) | Patients dropped out  (N=342) | t/χ2/U | *P* value |
| --- | --- | --- | --- | --- | --- |
| **Demographics** |  |  |  |  |  |
| Age [years, Mean ± SD] | 67.22±8.65 | 67.53±8.00 | 67.08±8.94 | -0.533 | 0.594 |
| Sex [female, n (%)] | 301 (60.56) | 92 (59.35) | 209 (61.11) | 0.138 | 0.711 |
| BMI [kg/m^2^, Mean ± SD] | 23.90±3.37 | 24.13±3.23 | 23.80±3.44 | -0.994 | 0.321 |
| Education [years, median (IQR)] | 11.00 (8.75, 13.00) | 12.00 (9.00, 13.00) | 11.00 (8.38, 12.13) | -0.955 | 0.340 |
| *APOE* ε4 status [yes, n (%)] | 169 (34.00) | 49 (31.61) | 120 (35.09) | 0.441 | 0.507 |
| Diagnosis [AD, n (%)] | 228 (45.88) | 63 (40.65) | 165 (48.25) | 2.481 | 0.115 |
| **Medical History** |  |  |  |  |  |
| Hypertension [yes, n (%)] | 216 (43.46) | 71 (45.81) | 145 (42.40) | 0.504 | 0.478 |
| Diabetes mellitus [yes, n (%)] | 92 (18.51) | 24 (15.48) | 68 (19.88) | 1.368 | 0.242 |
| Stroke [yes, n (%)] | 94 (18.91) | 31 (20.00) | 63 (18.42) | 0.173 | 0.677 |
| CHD [yes, n (%)] | 97 (19.52) | 34 (21.94) | 63 (18.42) | 0.839 | 0.360 |
| Hyperlipidemia [yes, n (%)] | 211 (42.45) | 72 (46.45) | 139 (40.64) | 1.473 | 0.225 |
| Smoking [yes, n (%)] | 102 (20.52) | 34 (21.94) | 68 (19.88) | 0.275 | 0.600 |
| Alcohol consumption [yes, n (%)] | 132 (26.56) | 44 (28.39) | 88 (25.73) | 0.386 | 0.535 |
| **Neuropsychological battery** |  |  |  |  |  |
| MMSE [scores, median (IQR)] | 24.00 (16.00, 27.00) | 24.50 (19.00, 27.00) | 23.00 (15.00, 27.00) | -3.329 | <0.001 |
| MoCA [scores, median (IQR)] | 18.00 (11.00, 22.00) | 19.00 (13.00, 24.00) | 17.00 (9.00, 22.00) | -3.615 | <0.001 |
| NPI [scores, median (IQR)] | 5.00 (0.00, 14.00) | 5.00 (0.00, 14.00) | 4.00 (0.00, 14.00) | -0.582 | 0.561 |
| ADL [scores, median (IQR)] | 21.00 (20.00, 29.00) | 21.00 (20.00, 26.00) | 21.00 (20.00, 31.00) | -1.113 | 0.266 |
| **Neuroimaging volume measures** |  |  |  |  |  |
| ChP [cm^3^, median (IQR)] | 2.22 (1.88, 2.63) | 2.25 (1.87, 2.63) | 2.19 (1.89, 2.64) | -0.268 | 0.788 |
| eTIV [cm^3^, Mean ± SD] | 1353.23 (1239.03, 1460.82) | 1354.28 (1249.93, 1449.61) | 1352.58 (1233.58, 1464.69) | -0.316 | 0.752 |
| Cortex [cm^3^, Mean ± SD] | 402.60±49.63 | 413.26±44.83 | 397.77±50.99 | -3.255 | 0.001 |
| Subcortical GM [cm^3^, Mean ± SD] | 50.48±6.09 | 51.31±5.44 | 50.10±6.33 | -2.061 | 0.040 |
| Hippocampus [cm^3^, median (IQR)] | 7.41 (6.26, 8.28) | 7.56 (6.47, 8.35) | 7.40 (6.15, 8.24) | -1.275 | 0.202 |
| Lateral ventricles [cm^3^, median (IQR)] | 27.40 (18.15, 40.46) | 27.71 (18.28, 40.26) | 27.12 (18.08, 40.55) | -0.016 | 0.988 |
| WMH [cm^3^, median (IQR)] | 2.68 (1.41, 5.45) | 2.83 (1.53, 5.90) | 2.67 (1.40, 5.19) | -0.680 | 0.497 |

*P* values < 0.05 were considered statistical significance, and variables were performed by χ² test, t-test, or Mann–Whitney U test. Data are shown as the mean ± standard deviation, medians with interquartile ranges (IQRs) or n (%).

Abbreviations: AD, Alzheimer’s disease; BMI, body mass index; *APOE* ε4, apolipoprotein E type epsilon 4; CHD, coronary heart disease; MMSE, Mini-mental State Examination; MoCA, Montreal Cognitive Assessment; NPI, Neuropsychiatric Inventory; ADL, Activities of Daily Living; ChP, choroid plexus; eTIV, estimated total intracranial volume; GM, grey matter; WMH, white matter hypointensities.

# References

1. Katzman R, Zhang MY, Ouang-Ya-Qu, et al. A Chinese version of the Mini-Mental State Examination; impact of illiteracy in a Shanghai dementia survey. *J Clin Epidemiol*. 1988;41(10):971-8. doi: 10.1016/0895-4356(88)90034-0

2. Yu J, Li J, Huang X. The Beijing version of the Montreal Cognitive Assessment as a brief screening tool for mild cognitive impairment: a community-based study. *BMC Psychiatry*. 2012;12:156. doi: 10.1186/1471-244X-12-156

3. Leung VP, Lam LC, Chiu HF, et al. Validation study of the Chinese version of the neuropsychiatric inventory (CNPI). *Int J Geriatr Psychiatry*. 2001;16(8):789-93. doi: 10.1002/gps.427
